# Non-Pulmonary Vein Triggers of Atrial Fibrillation Are Likely to Arise from Low-Voltage Areas in the Left Atrium

**DOI:** 10.1038/s41598-019-48669-1

**Published:** 2019-08-22

**Authors:** Shunsuke Kawai, Yasushi Mukai, Shujiro Inoue, Daisuke Yakabe, Kazuhiro Nagaoka, Kazuo Sakamoto, Susumu Takase, Akiko Chishaki, Hiroyuki Tsutsui

**Affiliations:** 1 Department of Cardiovascular Medicine, Kyushu University Graduate School of Medical Sciences, Fukuoka, Japan; 2 Department of Health Sciences, Graduate School of Medical Sciences, Kyushu University, Fukuoka, Japan

**Keywords:** Interventional cardiology, Outcomes research

## Abstract

The pathophysiology of non-pulmonary vein (PV) triggers of atrial fibrillation (AF) is unclear. We hypothesized that left atrial non-PV (LANPV) triggers are associated with atrial tissue degeneration. This study analyzed 431 patients that underwent catheter ablation (mean age 62 yrs, 303 men, 255 paroxysmal AF [pAF] patients). Clinical and electrophysiological characteristics of non-PV trigger were analyzed. Fifty non-PV triggers in 40 patients (9.3%) were documented; LANPV triggers were the most prevalent (n = 19, 38%). LANPV triggers were correlated with non-paroxysmal AF (non-pAF) (OR 3.31, p = 0.04) whereas right atrial non-PV (RANPV) triggers (n = 14) and SVC triggers (n = 17) were not. The voltage at the LANPV sites during SR was 0.3 ± 0.16 mV (p < 0.001 vs. control site). Low-voltage areas (LVAs) in the LA were significantly greater in non-pAF compared to pAF (14.2% vs. 5.8%, p < 0.01). RANPV trigger sites had preserved voltage (0.74 ± 0.48 mV). Long-term outcomes of patients with non-PV triggers treated with tailored targeting strategies were not significantly inferior to those without non-PV triggers. In conclusion, non-PV triggers arise from the LA with degeneration, which may have an important role in AF persistence. A trigger-oriented, patient-tailored ablation strategy considering LA voltage map may be feasible and effective in persistent/recurrent AF.

## Introduction

Previous studies indicate that most of the triggers of atrial fibrillation (AF) originate from the pulmonary veins (PVs), and thus PV antrum isolation (PVAI) is well accepted as a basic ablation strategy for AF^1,2^. However, it is known that non-pulmonary vein (non-PV) triggers also play an important role in the pathogenesis of AF^3–6^. The main causes of AF recurrence are reconnection of PVAI or linear ablation lines, existence of atrial arrhythmic substrates, and existence of non-PV triggers^7,8^. Atrial substrate-targeting strategies such as linear ablation and the ablation of complex fractionated atrial electrograms (CFAE) have been introduced to improve therapeutic outcomes of recurrent/intractable cases^9,10^. However, a recent prospective randomized trial could not demonstrate any benefit of substrate targeting in the left atrium (LA) in addition to PVAI in patients with persistent AF^10^. Important roles of triggers including non-PV sites in AF persistence have recently been recognized, and a therapeutic significance of trigger-targeting strategies has been reported in persistent AF^11–15^. However, the pathophysiology and etiology of non-PV triggers remain largely unknown.

It was reported that low-voltage areas (LVAs), revealed by voltage mapping, represent atrial scarring/fibrosis and are involved in the pathogenesis of refractory AF^16,17^. However, pathophysiological relationship between LVAs and non-PV triggers remains unknown. We hypothesized that the occurrence of non-PV triggers is associated with degeneration of atrial tissue and thus LVA. We thus evaluated the prevalence and electrophysiological properties of non-PV triggers by focusing on LVA in the LA.

## Methods

### Study population

This study enrolled 431 consecutive patients referred to our institution for AF ablation between January 2010 and December 2016. The definition of paroxysmal AF (pAF) and non-paroxysmal AF (non-pAF) followed the American College of Cardiology/American Heart Association/Heart Rhythm Society guidelines^18^. We retrospectively analyzed clinical and electrophysiological characteristics of the patients who had documented AF initiation from non-PV triggers. This study was in compliance with the principles outlined in the Declaration of Helsinki and was approved by the institutional review board for ethics at our institution, Kyushu University Hospital (approval no. 29–44). Informed consent was obtained in the form of opt-out on the web-site (https://www.cardiol.med.kyushu-u.ac.jp/research/clinical-research/).

### Electrophysiological study

Antiarrhythmic drugs were discontinued for at least five half-lives before the AF ablations. The presence of LA thrombi was excluded by contrast-enhanced computed tomography. When a contrast defect in the left atrial appendage was suspected, we excluded the presence of LA thrombi by transesophageal echocardiography. All patients underwent an electrophysiological study in the fasting state under deep sedation. A 20-pole catheter was inserted through the right jugular vein (BeeAT Japan Lifeline, Tokyo). The proximal portion of the catheter was positioned along the superior vena cava (SVC) and crista terminalis (CT), and the distal portion was positioned in the coronary sinus for pacing and internal cardioversion.

Following a trans-septal puncture under guidance with an intracardiac echocardiography catheter (5.5–10 MHz, 8Fr, AcuNav, Biosense Webster, Diamond Bar, CA), two or three long sheaths (SL1, AF Division, St. Jude Medical, Minneapolis, MN) were introduced into the LA via the same trans-septal puncture site. After left atriography was performed, mapping catheters (20-pole circular catheter or PentaRay NAV catheter, Biosense Webster, Diamond Bar, CA) and an ablation catheter were positioned in the pulmonary veins. The electrophysiological studies were performed under the support of an electroanatomical mapping system with the CARTO system (Biosense Webster) or Ensite Velocity system (St. Jude Medical).

### AF induction testing

An AF induction test was conducted before and after each PVAI procedure. When spontaneous AF firing was not observed, we tried to induce AF in one or both the following ways, as described:^11–14^ (1) induction with a bolus injection of isoproterenol (3–5 μg) and adenosine (20 mg). If not induced, (2) intentional defibrillation of AF induced by atrial burst pacing (30-beat at an amplitude of 10 V and pulse width of 1 ms from the ostium of the coronary sinus; increasing from 240 to 320 ppm in steps of 20 ppm or failure to 1:1 atrial capture) to evoke the immediate recurrence of AF. In case of an immediate recurrence of AF, the beat initiating AF was considered the trigger.

### Non-PV trigger mapping

When AF was induced and a non-PV trigger was suspected before PVAI procedure in first session cases, we proceeded with PVAI completion. In multiple session cases, we first evaluated the reconnections of PVAI lines in the previous sessions and re-ablated if reconnection sites existed.

AF induction test was repeated and only reproducible non-PV triggers were mapped. When AF was induced and a non-PV trigger was suspected after PVAI, mapping catheters were re-positioned in the atria, such as the left atrial posterior free wall, CT, or atrial septum according to the possible region of non-PV triggers estimated from the intracardiac activation pattern of the multipolar catheters located in both atria. Practically, when more than 2 separated sites were found as earliest activation sites in a formation of electrode catheters, we judged that the true earliest activation site existed somewhere else. Mapping catheters were re-positioned again and an AF induction test was repeated to identify the true earliest activation site with sufficient precedence (see Supplementary Fig. S1).

We recorded the voltage of non-PV trigger site during sinus rhythm and analyzed the local electrophysiological conduction properties at the onset of AF with mapping catheters and an ablation catheter. We also recorded the non-PV trigger sites by tagging onto a 3D electroanatomical mapping. Premature atrial contractions that did not initiate AF were excluded. The occurrence of non-PV triggers was analyzed in a patient-by-patient basis, including cases with multiple procedures.

### Quantitative analysis of the LA low-voltage areas

We depicted a whole LA voltage map during sinus rhythm using a duodecapolar catheter in a series of patients. The voltage height was measured as a bipolar peak-to-peak electrogram amplitude, and voltage values < 0.5 mV was defined as low voltage, as done in previous studies^16,17^. Each LVA was quantified and calculated by the ratio to the whole LA body surface area except the 4 PVs. We considered LVA with ≥10% of the LA body surface area as significant.

### Catheter ablation

We performed PVAI-based ablation in all patients. A 3.5-mm open-irrigated-tip ablation catheter was used (Navistar Thermocool, Biosense Webster, Diamond Bar, CA). Radiofrequency energy was delivered with a maximum temperature setting of 43 °C and a power of 20–35 W. We defined successful PVAI as the loss of PV potentials during sinus rhythm or coronary sinus pacing (entrance block) and local PV capture without left atrial capture by pacing from a circular mapping catheter or an ablation catheter placed at the PVs just distal to the radiofrequency ablation lesions (exit block).

In cases with documented non-PV triggers, we added ablation targeting the trigger sites and aimed at complete elimination of the AF inducibility from the targeted sites. Linear ablation (LA roof line/mitral isthmus line) was added after PVAI when an AF induction test induced LA roof dependent atrial tachycardia (AT)/mitral AT. A defragmentation ablation was added after PVAI in patients with prominent CFAE based on a CFAE map during AF rhythm.

### Follow-up

Patients were followed-up in our outpatient clinic at least at 1 month, 3 months, 6 months, and 12 months after the procedure during the first year and every 6 months afterwards. A clinical evaluation and a 12-lead surface ECG recording were performed at each visit. In addition, whenever patients experienced symptoms suggestive of an arrhythmia, they were instructed to visit our outpatient clinic or the referring physician in order to record a 12-lead surface ECG. In patients with permanent pace maker/intracardiac defibrillator, routine device interrogation was also performed to detect recurrent AF/AT. Holter recording (24 hour) was performed at least once during the period of 6 to 12 months after procedure by the referring physician. The first 3 months after the procedure were regarded as a blanking period for episodes, and arrhythmia recurrence was defined as the detection of AF/AT lasting >30 seconds after the blanking period. It was at the discretion of the treating physicians whether previously tolerated antiarrhythmic drugs were restarted or not after the catheter ablation. When restarted, antiarrhythmic drugs were typically discontinued at 3 months if patients had paroxysmal AF and at 12 months if patients had non-paroxysmal AF before ablations.

### Statistical analysis

The data are expressed as mean ± standard deviation (SD) for continuous variables, and counts and percentages for categorical variables. A comparison of categorical variables between pairs of groups was carried out using the chi-square test or Fisher’s exact test. A comparison of continuous variables between pairs of groups was carried out using Student *t*-test. We carried out a univariate analysis and a multivariate analysis of continuous variables between pairs of groups using a logistic regression analysis. Variables selected to be tested in the multivariate analysis were those with p-values < 0.1 in the univariate analysis. AF/AT-free survival over time was calculated by Kaplan-Meier method, and log-rank statistics were used for comparisons between groups. All tests were two-sided, and p-values < 0.05 were considered significant. Analyses were conducted using a software program JMP (SAS, Cary, NC). The power for the log-rank test was calculated using the bootstrap method that repeat simulating analyses for re-extracted datasets (SAS ver.14.0).

## Results

### Patient characteristics

A total of 431 patients with 512 ablation sessions were studied; 303 men (70.3%) and 128 women (29.7%), and mean age 62 ± 12 years. There were 255 pAF patients (59.2%) and 176 non-pAF patients (40.8%). Non-PV triggers were documented in 40 patients (9.3% of total). We divided the patients into 2 groups; non-PV trigger-positive group [nPV (+), n = 40] and non-PV trigger-negative group [nPV (−), n = 391].

Table 1 summarizes the baseline characteristics of the 2 groups. There were no significant differences in age and comorbidities such as diabetes mellitus, hypertension, structural heart disease, coronary artery disease, heart failure, and pacemaker/implantable cardioverter defibrillator (ICD) as well as the CHADS_2_ score between the 2 groups. AF type (pAF or non-pAF), left atrial diameter (LAD), left ventricular ejection fraction (LVEF) was also comparable between the 2 groups. Biomarkers such as brain natriuretic peptide (BNP), C-reactive protein (CRP) was also comparable between the 2 groups. The nPV (+) group had more female (50% vs. 27.6%; p = 0.003) and multiple ablation sessions (50% vs. 12.3%; p < 0.0001) compared to the nPV (−) group. In multiple ablation session cases, the percentage of PVAI line reconnection at least in one PV was 75% in nPV (+) group and 77% in nPV (−) group, respectively (N.S.).

**Table 1:** Patient characteristics.

Characteristics	nPV (−) (n = 391)	nPV (+) (n = 40)	*p*
Age (yrs)	62 ± 12	60 ± 17	0.38
Female	108 (27.6)	20 (50)	0.003
Diabetes mellitus	63 (16.1)	4 (10.0)	0.37
Hypertension	223 (57.0)	17 (42.5)	0.08
Structural heart disease	60 (15.4)	2 (5.0)	0.1
Coronary artery disease	29 (7.4)	2 (5.0)	0.76
Congestive heart failure	75 (19.2)	6 (15.0)	0.52
Pacemaker/ICD implantation	18 (4.6)	3 (7.5)	0.43
CHADS_2_ score	1.2 ± 1.1	0.9 ± 0.9	0.06
Non-paroxysmal AF (non-pAF)	159 (40.7)	16 (40.0)	0.91
LAD (mm)	41.3 ± 7.9	41.7 ± 8.1	0.79
LVEF (%)	64.5 ± 11.5	66.8 ± 7.9	0.23
BNP (pg/ml)	155 ± 319	90 ± 111	0.2
CRP (mg/dl)	0.23 ± 0.54	0.10 ± 0.14	0.15
Multiple ablation session	48 (12.3)	20 (50.0)	<0.0001

BNP: brain natriuretic peptide, CRP: C-reactive protein, ICD: implantable cardioverter defibrillator, LAD: left atrial diameter, LVEF: left ventricular ejection fraction.

### Prevalence and distribution of non-PV triggers

At the beginning of ablation procedure, 161 out of 512 sessions (31.9%) were in AF while other 351 were on sinus rhythm. In this study, 168 patients had more than one detectable trigger (PV trigger or non-PV trigger or both) (39%), and 263 patients did not have any detectable triggers (61%). In 168 patients who had at least one trigger, 22 patients had only non-PV triggers (13.1%), 128 patients had only PV triggers (76.2%), and 18 patients had both non-PV triggers and PV triggers (10.7%). We documented a total of 232 triggers; 182 PV triggers (78% of the total) and 50 non-PV triggers (22%). The PV trigger sites were in the left superior pulmonary vein (n = 85; 47%), left inferior pulmonary vein (n = 38; 21%), right superior pulmonary vein (n = 53; 29%) and right inferior pulmonary vein (n = 6; 3%). The distribution of PV trigger sites was mostly the same as in previous reports^1^.

Fifty non-PV triggers were documented in 40 patients (9.3% of total). Repetitive spontaneous or drug-induced AF firing and termination was observed in 27 patients (67.5%) where the initial ectopic beats were mapped. In 13 patients with AF persistence after induction (32.5%), 3.8 ± 2.8 times electrical cardioversions were required to complete trigger mappings. In the nPV (+) group, 18 patients had both PV triggers and non-PV triggers (45%), and 10 patients had more than 2 non-PV triggers (25%). Location of the non-PV trigger sites were in the SVC (n = 17; 34%), left atrial posterior free wall (n = 16; 32%), left atrial septum (n = 3; 6%), right atrial CT (n = 8; 16%) and ‘other’ right atrium (n = 6; 12%). Thus, the most prevalent location outside the venous system was in the LA.

### Clinical characteristics in each non-PV trigger group

We compared the clinical backgrounds of each non-PV trigger group of SVC, RA and LA with those of nPV (−) group (Table 2). The univariate analysis revealed that female gender was significantly correlated with all the groups of non-PV triggers (p = 0.03, 0.006 and 0.07, respectively). Pacemaker/ICD implantation was correlated with non-PV triggers in the LA (p = 0.04) but not with non-PV triggers in the SVC and RA. Non-pAF was significantly correlated with non-PV triggers in the LA (p = 0.02) but not with non-PV triggers in the SVC and RA. LVEF was significantly correlated with non-PV triggers in the SVC (p = 0.04) but not with non-PV triggers in the RA and LA. Multiple ablation session was correlated with non-PV triggers in the RA and LA (p = 0.0004 and p < 0.0001, respectively) but not with non-PV triggers in the SVC. The multivariate analysis revealed that female gender and multiple ablation session were significantly correlated with the presence of non-PV triggers as a whole (OR 2.85 and 7.69, p = 0.004 and p < 0.0001, respectively) (Table 3). Female gender was significantly correlated with non-PV triggers in the RA (OR 5.83, p = 0.004) and tended to be correlated with non-PV triggers in the SVC and LA but not with statistical significance (p = 0.06, each). Multiple ablation session was significantly correlated with non-PV triggers in the RA and LA (OR 9.53 and 25.3, p = 0.0001 and p < 0.0001, respectively) but not with non-PV triggers in the SVC (p = 0.21). Non-pAF was significantly correlated with non-PV triggers in the LA (OR 3.31, p = 0.04) and tended to be inversely correlated with non-PV triggers in the SVC and RA but with no statistical significance (OR 0.65 and 0.71, p = 0.47 and p = 0.61, respectively). Subsequently, the prevalence of non-pAF was significantly different among the three groups (LA non-PV trigger group: 68%, RA non-PV trigger group: 29%, and SVC non-PV trigger group: 24%) (p = 0.01).

**Table 2:** Univariate analysis for correlation with non-PV triggers.

Characteristics	nPV (−) (n = 391)	SVC (+) (n = 17)	*p*	RA (+) (n = 14)	*p*	LA (+) (n = 19)	*p*
Age (yrs) (per decade)	62 ± 12	62 ± 17	0.91	59 ± 17	0.26	61 ± 17	0.57
Female	108 (27.6)	9 (52.9)	0.03	9 (64.3)	0.006	9 (47.4)	0.07
Diabetes mellitus	63 (16.1)	2 (11.8)	0.63	1 (7.1)	0.38	2 (10.5)	0.52
Hypertension	223 (57.0)	8 (47.1)	0.42	6 (42.9)	0.3	8 (42.1)	0.21
Structure heart disease	60 (15.4)	1 (5.9)	0.31	0 (0)	—	2 (10.5)	0.57
Coronary artery disease	29 (7.4)	1 (5.9)	0.81	0 (0)	—	2 (10.5)	0.62
Congestive heart failure	75 (19.2)	1 (5.9)	0.2	2 (14.3)	0.65	4 (21.1)	0.84
Pacemaker/ICD implantation	18 (4.6)	1 (5.9)	0.81	0 (0)	—	3 (15.8)	0.04
CHADS_2_ score	1.2 ± 1.1	0.9 ± 1.0	0.26	0.9 ± 1.0	0.38	0.9 ± 0.8	0.25
Non-paroxysmal AF (non-pAF)	159 (40.7)	4 (23.5)	0.16	4 (28.6)	0.36	13 (68.4)	0.02
LAD (mm)	41.3 ± 7.9	41.6 ± 7.6	0.89	40.0 ± 8.1	0.49	42.9 ± 8.7	0.4
LVEF (%)	64.5 ± 11.5	70.3 ± 5.4	0.04	67.2 ± 7.2	0.39	64.3 ± 9.4	0.94
BNP (pg/ml)	155 ± 319	56 ± 47	0.11	71 ± 71	0.25	135 ± 143	0.81
CRP (mg/dl)	0.23 ± 0.54	0.08 ± 0.09	0.23	0.07 ± 0.1	0.19	0.14 ± 0.18	0.49
Multiple ablation session	48 (12.3)	4 (23.5)	0.18	7 (50)	0.0004	15 (78.9)	<0.0001

**Table 3:** Multivariate analysis for correlation with non-PV triggers.

	Non-PV All (n = 40)			SVC (n = 17)			RA (n = 14)			LA (n = 19)		
	OR	95% CI	p	OR	95% CI	p	OR	95% CI	p	OR	95% CI	p
Female	2.85	1.39–5.88	**0.004**	2.6	0.94–7.3	0.06	5.83	1.83–20.93	**0.004**	2.87	0.95–8.75	0.06
Pacemaker/ICD	1.38	0.26–5.43	0.67	1.25	0.06–8.12	0.84	—	—	1	3.04	0.49–16.32	0.2
Non-pAF	1.07	0.50–2.23	0.86	0.65	0.17–1.98	0.47	0.71	0.17–2.47	0.61	3.31	1.1–11.18	0.04
LVEF (per 10)	1.24	0.89–1.78	0.22	1.82	1.04–3.54	0.05	1.06	0.63–1.99	0.83	1.11	0.72–1.79	0.65
Multiple ablation session	7.69	3.75–16.0	**<0.0001**	2.19	0.57–6.83	0.21	9.53	2.96–31.64	**0.0001**	25.3	8.5–93.57	**<0.0001**

### The electrophysiological properties of the non-PV trigger sites

**Figure 1:**
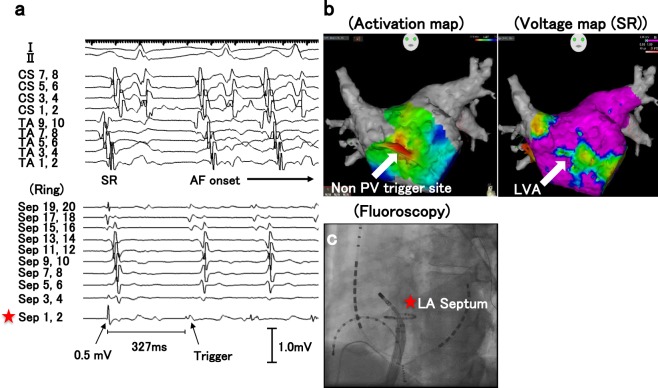
A representative case with a non-PV trigger from the LA septum. (**a**,**b)** The intracardiac electrograms and electroanatomical mapping indicate that the non-PV trigger arises from an LVA. (**c**) The fluoroscopy shows the catheter position. Red asterisks indicate the trigger site of AF. CS: coronary sinus, TA: tricuspid annulus.

**Figure 2:**
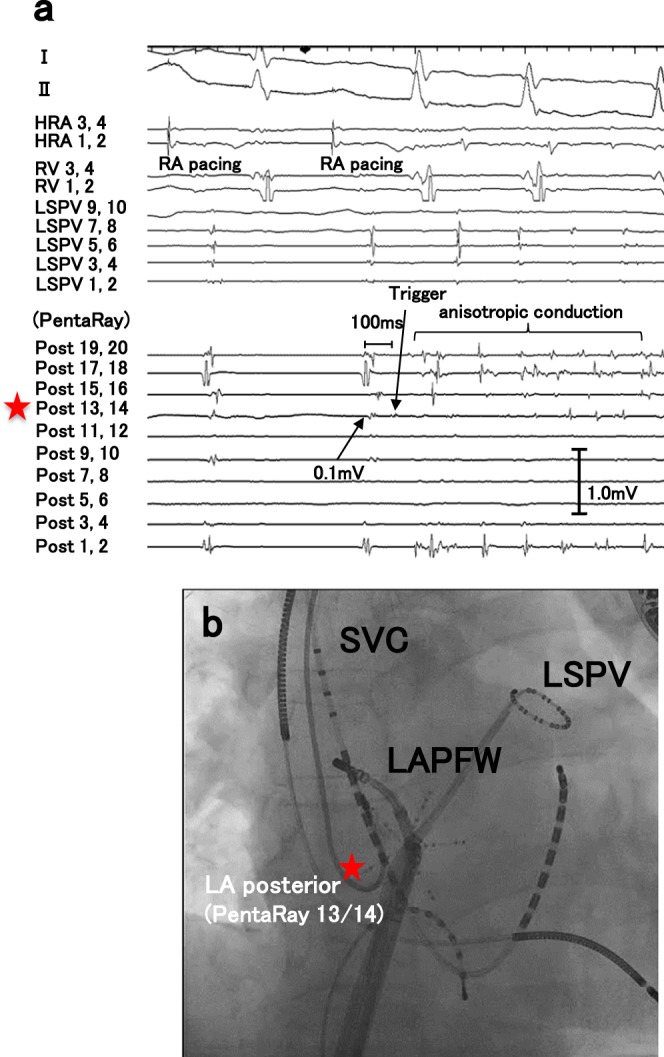
A representative case with a non-PV trigger from the left atrial posterior free wall. (**a**) Delayed and anisotropic conduction following an ectopic trigger is observed at AF initiation. (**b**) Red asterisks in the fluoroscopies indicate AF trigger site. HRA: high right atrium, LAPFW: left atrial posterior free wall, LSPV: left superior pulmonary vein, SVC: superior vena cava, RV: right ventricle.

**Figure 3:**
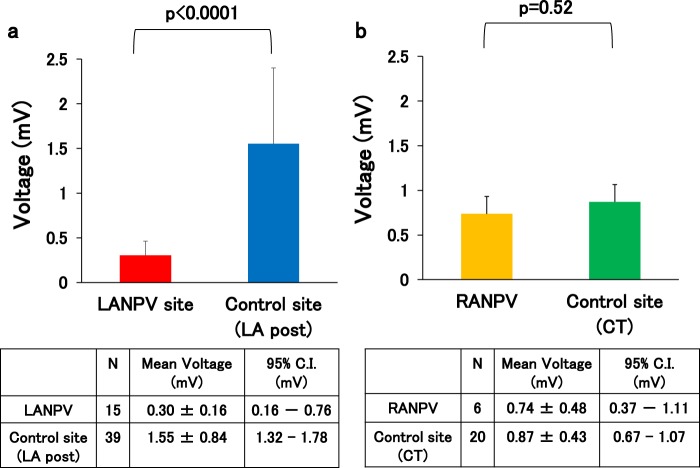
Voltage of non-PV trigger sites during sinus rhythm. (**a**) The voltage of LANPV sites was lower than that of control sites (LA posterior wall). (**b**) The voltage of RANPV sites was preserved compared to that of control sites (crista terminalis). CT: crista terminalis.

Representative intracardiac electrograms at the onset of AF from LANPV triggers were shown in Figs 1 and 2. The activation map and the voltage map showed that the ectopic beat that initiated AF existed within LVAs (Fig. 1). In some cases, delayed and anisotropic conductions around the initial ectopic site could be observed at the AF onset (Fig. 2). The mean voltage of the 19 LANPV sites during sinus rhythm was 0.3 ± 0.16 mV, and 93.3% of the voltage values of LANPV sites were ≤0.5 mV (Fig. 3).

We have validated three control measurements for the LA respectively; atrial voltage in the coronary sinus in the 15 patients with LANPV triggers (1.69 ± 1.23 mV), atrial voltage in the LA posterior wall in the 39 patients without non-PV triggers (1.55 ± 0.84 mV), and whole LA atrial voltage in the 19 pAF patients without non-PV triggers (1.13 ± 0.34 mV) as a relatively healthy control. Every comparison showed a similar result that the voltage at the LANPV trigger is significantly lower than that in each control data. Therefore, we regarded the atrial voltage in the LA posterior wall of the patients in the nPV (−) group as a control site because the majority of the LANPV trigger sites were located in the LA posterior wall (n = 16/19; 84.2%). Consequently, The mean voltage of the control sites was 1.55 ± 0.84 mV. The voltage values of the LANPV sites were significantly lower than those of the control sites (p < 0.0001) (Fig. 3a). We also evaluated the local voltage in the nPV (+) patients of the LA posterior trigger site (n = 13) and the mean voltage was 0.32 ± 0.16 mV. For this specific group, the comparison with control LA posterior wall showed a similar result (p < 0.0001). The RANPV trigger sites were mainly the CT. The mean voltage during sinus rhythm at the RANPV sites was 0.74 ± 0.48 mV. We regarded the atrial voltage in the CT of the pAF patients in the nPV (−) group as a control site because the majority of the RANPV trigger sites located in the CT (n = 8/14; 57.1%), and the mean voltage of the control sites was 0.87 ± 0.43 mV. The voltage values of the RANPV sites were not significantly different from those of the control cases (p = 0.52) (Fig. 3b). As with the LANPV trigger, we also evaluated the local voltage in a specific group with the CT trigger sites (n = 4) and the mean voltage for them was 0.67 ± 0.34 mV. The comparison with control CT value showed no significant difference (p = 0.39).

### Quantitative analysis and characterization of LVAs in the LA

**Figure 4:**
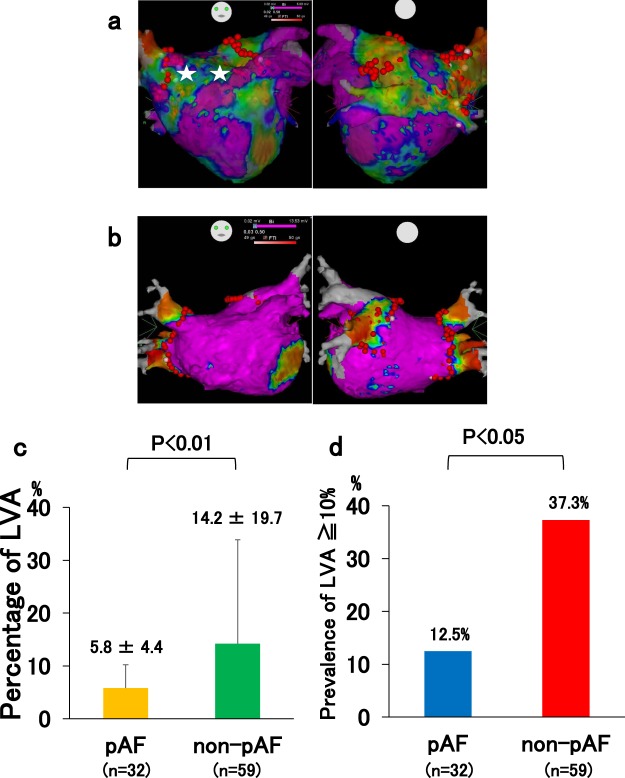
The relationship between LVAs and the AF type. (**a**) LVA-positive case with persistent AF; the percentage of LVA was 24.6%. Two asterisks indicate AF trigger (non-PV) sites in the LVA. (**b**) LVA-negative case of pAF; the percentage of LVA was <10%. (**c**) Mean percentage of LVA in the pAF and non-pAF groups. (**d**) Prevalence of LVA ≥ 10% in these two groups.

In consideration of the significant relationship among LANPV, non-pAF and LVA, we obtained whole LA voltage maps during sinus rhythm in a group of patients (n = 91: 32 pAF patients and 59 non-pAF patients) (Fig. 4). The mean percentage of LVA in the non-pAF patients was significantly greater than that in the pAF patients (14.2 ± 19.7% vs. 5.8 ± 4.4%, p < 0.01) (Fig. 4c). The total number of patients with the percentage of LVA ≥ 10% was 26 (28.6%). The prevalence of LVA ≥ 10% in the non-pAF patients was significantly greater than that in the pAF patients (37.3% vs. 12.5%, p < 0.05) (Fig. 4d). The mean left atrial volume index in the patients with LVA ≥ 10% tended to be larger than that in the patients with LVA < 10%, but the difference was not significant (48 ± 16.1 ml/m^2^ vs 42 ± 13.4 ml/m^2^, p = 0.12).

### Acute and long-term outcomes in patients with non-PV triggers

**Figure 5:**
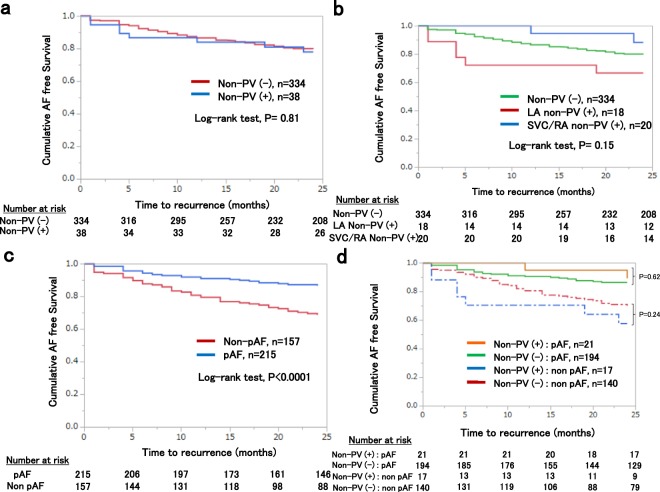
Kaplan-Meier curves showing AF/AT recurrence-free survival after the last ablation. (**a**) AF/AT-free survival rate in patients with non-PV triggers and without. (**b**) AF/AT-free survival rate in patients with LA non-PV triggers, SVC/RA non-PV triggers and in the control group respectively. (**c**) AF/AT-free survival rate in patients with pAF and non-pAF. **(d**) AF/AT-free survival rate in patients with or without non-PV triggers in pAF and non-pAF separately.

Completion of PVAI was achieved in all session. Ninety-two patients (23.5%) in the non-PV (−) group and 7 patients (17.5%) in the non-PV (+) group underwent line ablation, and the difference was not significant (P = 0.43). Also, 46 patients (11.8%) in the non-PV (−) group and 5 patients (12.5%) in the non-PV (+) group underwent defragmentation, and the difference was not significant (P = 0.8). The documented non-PV triggers were ablated in 47 out of the 50 sites and 46 of them were eliminated, confirmed by a non-inducibility (97.9%). In 3 patients with SVC trigger, non-PV trigger ablation was not performed because non-PV trigger was induced before the completion of PVI but was not induced after the completion of PVI. In a patient with LA septum trigger, the trigger foci could not be totally eliminated due to wondering of the earliest sites. Long-term follow-up data could be obtained from 372 out of 431 patients (86.3%); 334 patients (85.4%) in the non-PV (−) group and 38 patients (95%) in the non-PV (+) group. Mean follow-up period after the last procedure was 28.2 ± 16.4 months. A hundred and sixty-five patients (49.4%) in the non-PV (−) group and 18 patients (47.4%) in the non-PV (+) group continued antiarrhythmic drugs throughout the follow-up period. During the study period, 101 patients (23.4%) had recurrent AF. A Kaplan-Meier survival analysis revealed that AF recurrence rate after the last procedure was not significant different between the non-PV (+) and the non-PV (−) groups (P = 0.81, power of non-significance = 0.922) (Fig. 5a). The AF-free survival rate at 2 yrs. and the 95% confidence interval in the non-PV (+) group were 0.78 and 0.61–0.88, whereas those in the non-PV (−) group were 0.79 and 0.74–0.83, respectively, with no statistical difference.

We next classified all patients into 3 groups [non-PV (−) group (N = 334), LA non-PV (+) group (N = 18) and SVC/RA non-PV (+) group (N = 20)] to compare long-term outcomes. The Survival rate free from AF/AT recurrence in each group was 76.1%, 66.7% and 87.5% at 24 months of follow-up, respectively. A Kaplan-Meier survival analysis revealed that AF recurrence were not significantly different among the 3 groups although the LA non-PV group tended to have poorer prognosis (P = 0.15, power of non-significance = 0.64) (Fig. 5b). In overall, non-pAF patients had significantly higher AF recurrence rate compared to pAF patients (Fig. 5c). In consideration of prevalent LA non-PV triggers in non-pAF patients, we analyzed the outcome of non-PV (+) patients in patients with pAF and non-pAF separately (Fig. 5d). Consistently, AF recurrence rate was not significantly different in non-PV (+) patients compared to the non-PV(−) patients both in pAF and non-pAF populations although non-PV (+) patients with non-pAF tended to have poorer prognosis without statistical significance (P = 0.24).

## Discussion

### Main findings

The main findings of this study are as follows; (1) Non-PV triggers were documented in 9.3% of the patients undergoing AF ablation, (2) Non-PV triggers were significantly prevalent in female patients, (3) LANPV triggers had a strong correlation with non-pAF, whereas non-PV triggers from the SVC and the RA had no correlation with AF type, (4) LANPV triggers were associated with regional LVAs whereas RANPV triggers were not, (5) Low-voltage areas in the LA were significantly prevalent and more extensive in non-pAF patients compared to pAF, and (6) Long-term outcome in patients with non-PV triggers treated with tailored trigger ablation strategies was not significantly different to those without non-PV triggers.

To the best of our knowledge, this is the first report that demonstrates a direct relationship between non-PV triggers of AF and low voltage area in the LA, and thus the degeneration of atrial wall.

### The prevalence and distribution of non-PV triggers

Non-PV triggers play an important role in a significant portion of AF patients^3–5^. It has been reported that non-PV triggers emerge from the SVC, left atrial posterior free wall, CT, coronary sinus ostium, ligament of Marshall, left atrial appendage, and interatrial septum^3–5^. In our study, the non-PV trigger sites were the SVC (n = 17, 34%), left atrial posterior free wall (n = 16, 32%), left atrial septum (n = 3, 6%), CT (n = 8, 16%) and other RA (n = 6, 12%). Thus, LANPV triggers were the most common non-PV triggers. In terms of embryology, “the sinoatrial node is derived from the sinus venosus”, and the other remnants of the embryonic sinus venosus are present in several areas including the musculature of the SVC, coronary sinus, PV, and CT^19,20^. These areas are well-known arrhythmogenic sites and possible trigger sites of AF. The presence of non-PV triggers was reported to be relevant to clinical characteristics such as gender and LA enlargement^4^. Our present findings also demonstrated that female gender was significantly correlated with the presence of non-PV triggers, especially in the group of RANPV. These findings are compatible with a previous report about pAF^4^.

Our results also demonstrated that LANPV triggers were significantly associated with non-PAF, whereas non-PV triggers from the RA and SVC were not associated with non-PAF. It is suggested that LANPV triggers are different from non-PV triggers from the RA and SVC in their pathogenesis, and are more closely associated with the disease progression of AF.

In contrast to previous studies^4,16^, our findings did not demonstrate a significant relationship between LANPV triggers and LA enlargement. However, it is conceivable that electrical remodeling and arrhythmogenesis of the LA might progress in a localized area and precede LA enlargement^21^. Supporting this idea, the present study demonstrated that the existence of LVA ≥ 10% had no significant correlation with LA enlargement.

### LANPV triggers and low-voltage areas

Low-voltage areas were reported to be relevant to atrial fibrosis and scarring^17^. Verma *et al*. showed that pre-existent left atrial scarring was a strong predictor of clinical failure after PVAI^16^, suggesting that LVAs have arrhythmogenic properties and are involved in the refractoriness and persistence of AF. In the present study, LANPV triggers existed in or around LVA. Previous studies showed that myofibroblasts depolarize cardiomyocytes by heterocellular electronic interactions via gap junctions and induce ectopic activity in fibrotic remodeled myocardium^22–24^. Thus, it may be possible that de novo ectopies are likely to emerge from degeneration of atrial wall that we observe as LVAs.

### LANPV triggers and the persistence of AF

Our results showed that LANPV triggers were strongly correlated with non-PAF. The cause and effect relationship of LANPV triggers and non-PAF has not been clearly demonstrable to date, it is well expected that LANPV triggers are involved in the disease progression of AF^25,26^. Interestingly, our present findings demonstrated that LVAs were more prevalent in non-pAF patients than in pAF patients. Taken together, our results suggest that LVAs act not only as an arrhythmogenic substrate but also have an important role as an origin of non-PV triggers and thus accelerate AF progression and persistence. On the other hand, non-PV triggers from the RA were located at the sites with preserved voltage and not associated with non-pAF in the present study. In addition, a previous study reported that LVAs in the RA were not extensive in non-pAF patients^27^. Thus, non-PV triggers from the RA may not have a significant correlation to disease progression of AF and/or degeneration of the atrial wall at least in the studied population.

### Long-term clinical outcome after non-PV trigger ablation

Previous studies indicated that unsuccessful mapping and ablation of non-PV triggers were associated with extremely high recurrence rate of AF^5,11^. These results indicated clinical importance of non-PV triggers and that residual non-PV triggers had roles in AF recurrence. In our study, long-term clinical outcomes of patients with non-PV triggers were not significantly different from those without non-PV triggers when treated with tailored targeting strategies although it is possible that difference among the groups could not be detected due to a small sample size.

Taken together, a targeted ablation of non-PV triggers is a feasible and effective therapeutic strategy in patients with non-PV triggers or with recurrent/intractable AF profile. It should be noted that clinically-relevant non-PV triggers would be undetectable unless intentional AF induction and trigger mapping protocol were performed as a strategy. In terms of the locations of non-PV triggers, patients with LA non-PV triggers had the worst recurrence rate although not statistically significant in the relatively small number of the studied population. In contrast, patients with non-PV triggers in the RA/SVC had an excellent clinical outcome when treated with tailored targeting strategies.

### Clinical implications

Our results indicate that LVA mapping may provide important landmarks when searching for non-PV triggers during an ablation procedure, which helps optimal mapping procedure and therapeutic design. Several recent studies showed favorable results with LVA ablation in terms of substrate modification in addition to PVAI^28–31^. Their results may in part be due to the elimination of non-PV triggers in LVAs. Furthermore, clinical importance of non-PV trigger elimination should be reconsidered in patients with persistent or recurrent AF^12–15^. It remains to be clarified whether a trigger-based ablation strategy focusing on LVA is more feasible and effective compared to an ablation strategy targeting LVAs as a crude.

### Limitations

There were several limitations in our study. First, this was a single-center, observational study. Therefore, the sampling size was small and relatively underpowered. The present findings need to be confirmed in multi-center prospective studies. Second, the triggers induced in the electrophysiological studies might not necessarily represent the true clinical triggers, and vice versa. We also might have underscored true non-PV triggers because of non-inducibility or poor reproducibility at the time of electrophysiological studies. However, our results showed that the clinical outcomes of patients with non-PV triggers were acceptable compared to previous studies, implicating successful ablation of clinically-relevant AF triggers in our studied patients. Third, we quantified LVAs only in the LA, and could not evaluate the entire RA. It is conceivable that a de-novo arrhythmogenicity may be acquired in a degenerated RA in specific populations. Fourth, asymptomatic AF recurrence might have been overlooked in the follow-up method in our study, which might have influenced the long-term outcome data. Finally, LVA documented in the endocardial mapping could not reflect all atrial scarring/fibrosis. Degeneration of atrial wall at the epicardial side might participate the arrhythmogenicity of the atria as suggested in the studies using MRI^17^. Indeed, endocardial regional voltage was preserved in some LANPV cases in the present study. Further studies are needed to elucidate the pathophysiological relationship between non-PV triggers and degeneration of atrial wall.

## Conclusions

Non-PV triggers arise from LA with degeneration, which may have an important role in AF persistence. RANPV triggers may be more relevant to anatomical and embryological arrhythmogenicity compared to LANPV triggers. Clinical outcomes of patients with non-PV triggers were acceptable after tailored mapping and ablation. A trigger-oriented, patient-tailored ablation strategy considering a voltage map may be feasible and effective in persistent or recurrent AF.

## Supplementary information

Dataset 1

## Data Availability

All data generated or analyzed during this study are included in this published article and its Supplementary Information Files.
