# Supplementary material for: Non-Pulmonary Vein Triggers of Atrial Fibrillation Are Likely to Arise from Low-Voltage Areas in the Left Atrium
Source: Sci Rep. 2019 Aug 22;9:12271. doi: 10.1038/s41598-019-48669-1 (PMC6706423; doi:10.1038/s41598-019-48669-1)
Supplement: Supplementary file 1 — Dataset 1 [file 41598_2019_48669_MOESM1_ESM.pdf]

Non-Pulmonary Vein Triggers of Atrial Fibrillation Are Likely to Arise from Low-Voltage Areas in the Left Atrium

Shunsuke Kawai, MD; Yasushi Mukai, MD, PhD; Shujiro Inoue, MD, PhD; Daisuke Yakabe, MD; Kazuhiro Nagaoka, MD, PhD; Kazuo Sakamoto MD, PhD; Susumu Takase, MD, PhD; Akiko Chishaki, MD, PhD; Hiroyuki Tsutsui, MD, PhD

Supplementary Figure S1.

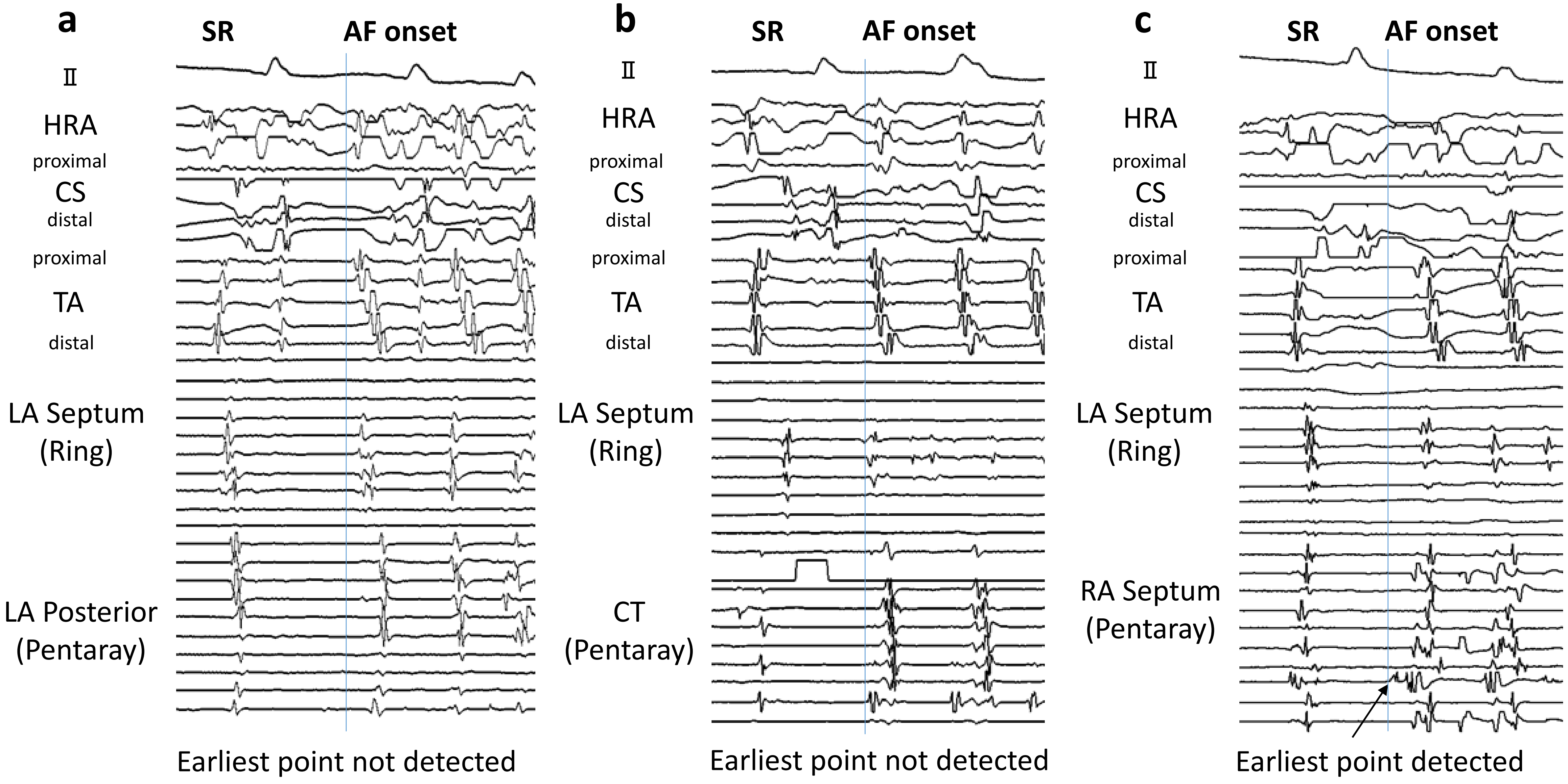

d e

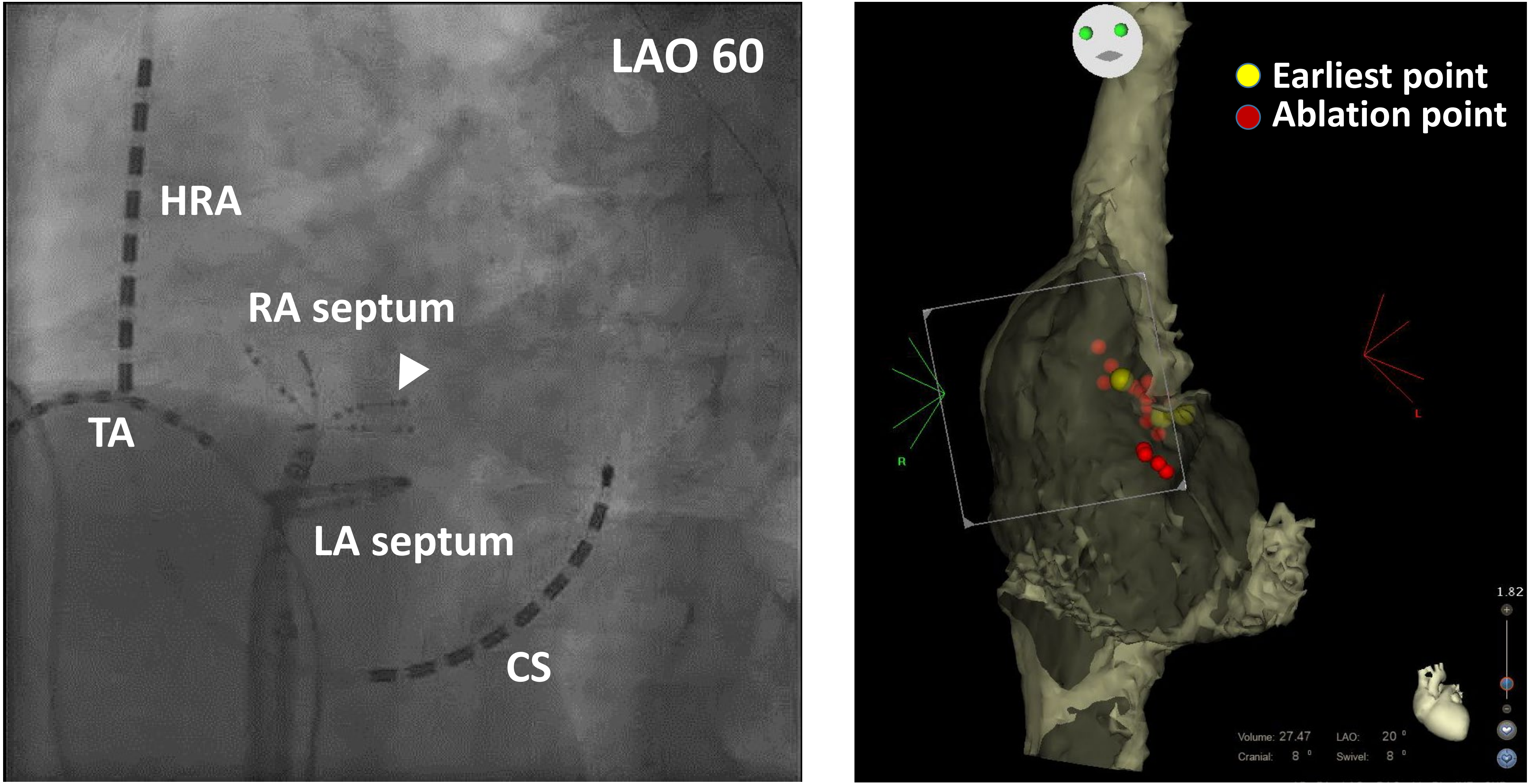
LAO 60
HRA
RA septum
TA
LA septum
CS
Earliest point
Ablation point
R
L
1.82
Volume: 27.47
LAO: 20 °
Cranial: 8 °
Swivel: 8 °

Supplementary Figure Legend

Fig. S1.

An example of non-PV trigger mapping procedure. In this case, a non-PV trigger arose from RA septum following intentional defibrillation of AF repetitively.

a; A 20 pole electrode ring catheter and Pentaray® NAV are positioned at LA septum and LA posterior wall, respectively. Blue line indicates AF onset. The earliest firing point is not detected in this electrode formation showing similar precedence in HRA, TA proximal and LA septum. It was suggested that the earliest firing point is within the triangle formed by the 3 regions. **b;** Pentaray® NAV is re-positioned to CT. The earliest firing point is still not detected but CT and LA septum showed similar precedence. **c;** Pentaray® NAV is again re-positioned to RA septum. The earliest firing point is eventually detected with sufficient precedence. **d;** Fluoroscopic view of the electrode formation. Arrowhead indicates the earliest firing point. **e;** The earliest firing points are tagged onto the 3D electroanatomical mapping with yellow dots. Reproducible non-PV trigger firing was confirmed three times.

CS: coronary sinus, CT: crista terminalis, HRA: high right atrium, LA: left atrium, LAO:

left anterior oblique, RA: right atrium, TA: tricuspid annulus.
